# Ecological and evolutionary influences on the elemental composition of birds

**DOI:** 10.1098/rspb.2025.1276

**Published:** 2025-10-08

**Authors:** Ethan S. Duvall, Louis A. Derry, Peter B. McIntyre, Alexander S. Flecker

**Affiliations:** ^1^ Department of Ecology and Evolutionary Biology, Cornell University, Ithaca, NY, USA; ^2^ Department of Earth and Atmospheric Sciences, Cornell University, Ithaca, NY, USA; ^3^ Department of Natural Resources, Cornell University, Ithaca, NY, USA

**Keywords:** ecological stoichiometry, body stoichiometry, elemental composition, avian ecology, bird biology, bird physiology, fat storage, body composition, zoogeochemistry, vertebrates

## Abstract

The evolutionary and ecological diversity of animals is often reflected in the elemental composition of their bodies. Despite decades of stoichiometric research, remarkably little is known about the elemental composition of birds, the most diverse group of land vertebrates. This gap limits our understanding of vertebrate body composition and its implications for ecosystem functioning. Here, we report the body stoichiometry (%C, %N, %P, C:N, C:P, N:P) of 32 bird species spanning diverse ecological traits and phylogenetic lineages. Compared to other vertebrates, birds exhibit consistently low phosphorus content, probably reflecting two key flight adaptations: skeletal minimization (i.e. restricted investment in phosphorus-rich bone) and feather production (i.e. investment in phosphorus-poor keratin). Among birds, carbon content is associated with body fat, which has distinct stoichiometry and is known to fluctuate seasonally. Feathers constitute ~25% of a bird’s body nitrogen on average, hence variation in feather investment can produce significant differences. Unlike patterns observed in other vertebrates, body size, taxonomy, phylogeny and diet poorly predict bird stoichiometry. Instead, we infer that selective constraints arising from flight (skeletal minimization, feather investment) and phenological cycles (fat storage, feather molting) shape bird stoichiometry. These findings can inform research on avian nutrition, ecology and zoogeochemistry amid global change.

## 1. Introduction

The remarkable diversity of animal ecology and evolutionary lineages is often mirrored in the elemental composition of their bodies. Accordingly, a key theme of ecological stoichiometry—the study of how imbalances in elemental availability affect ecological processes—is understanding how the elemental composition of organisms responds to and shapes interactions between animals and their environment [1]. For example, research on aquatic animals has shown that differences in body stoichiometry often reflect environmental constraints [2,3], drive variation in nutrient release [4–6] and govern contributions to ecosystem nutrient cycles [7]. Thus, data on the elemental composition of abundant animal species are essential for understanding how biodiversity loss under global change may affect ecosystem functioning.

Comparisons of body carbon (C), nitrogen (N) and phosphorus (P) across major animal groups show considerable between- and within-group variation [8]. Ecological stoichiometry theory interprets these patterns as reflections of imbalances among elements required for growth, metabolism and structural integrity [1]. In invertebrates, for example, body stoichiometry differences have been linked to growth-dependent allocation of P in ribosomal RNA [2] and structural allocation of N in chitinous exoskeletons, both of which are influenced by taxonomic identity, body size and trophic position [9,10]. In vertebrates, the presence of internal skeletons requires sequestration of bone-associated elements such as P and calcium (Ca), but variation also arises among taxonomic groups [4,11] and body sizes [12–14]. Nonetheless, even in well-studied taxa like fishes, predicting patterns of body stoichiometry from ecological traits or phylogenetic affinities remains challenging. For other vertebrates, predictive power is even more limited because the influence of environmental, physiological and evolutionary differences has not been tested.

Despite their diversity, mobility and contributions to elemental cycles globally [15–18], information on the elemental composition of birds remains scarce [8,19]. This lack of bird stoichiometry data impedes our ability to accurately estimate or predict avian contributions to elemental cycles. For example, previous analyses have relied on the assumption that nutrient release by birds primarily reflects dietary inputs rather than demands for building and maintaining body tissues [15,16]. However, recent research suggests that body stoichiometry differences arising from allometry and diet may help explain interspecific variation in nutrient release by vertebrates [20,21]. Additionally, the absence of bird stoichiometry data has hindered quantification of their nutrient transport at broad scales through movement and mortality [22]. These processes could be ecologically significant, especially in major migratory corridors or during mass mortality events [23,24], and might reveal hidden consequences of bird population declines [25].

Here, we examine variation in the elemental content (%C, %N, %P) and stoichiometric ratios (C:N, C:P, N:P) of 32 bird species, representing 22 families and 9 taxonomic orders collected in New York, USA (electronic supplementary material, table S1). Our whole-body analyses encompassed a 1000-fold range in body mass and spanned a wide diversity of phylogeny, diet and life history—from ruby-throated hummingbird (*Archilochus colubris*) to ruffed grouse (*Bonasa umbellus*), and Cooper’s hawk (*Accipiter cooperii*) to redhead duck (*Aythya americana*). We also assessed the elemental composition of major bird tissues, including fat, feathers, bone, muscle and organs (electronic supplementary material, table S2). With these data, we test a central premise of the ecological stoichiometry of vertebrates: that variation in body composition reflects differential investment in C-rich energy storage molecules (fats), N-rich tissues (muscle and feathers) and P-rich bones that in turn reflect evolutionary, ecological and physiological constraints. We specifically address four key questions for which results from birds help to expand the predictive power of ecological stoichiometry theory:

(1) *How distinct are the elemental compositions of different bird tissues?* We predicted that differences in the biochemical composition of bird tissues would give rise to contrasting elemental composition—with fat being C-rich, muscle and feathers being N-rich, and bone being P-rich—setting the stage for interpretation of differences in whole-body stoichiometry in terms of alternative tissue investment strategies.
(2) *How does the elemental composition of birds compare to other vertebrates?* We predicted that birds exhibit lower body %P and higher C:P and N:P ratios compared to non-flying vertebrates due to anatomical adaptations for flight—specifically, restricted investment in P-rich skeletal tissues and high allocation to P-poor feathers.
(3) *To what extent do birds vary taxonomically or phylogenetically in elemental composition?* We predicted that taxonomic and phylogenetic relationships have limited influence on bird body stoichiometry because shared requirements for flight (e.g. minimized skeletons, feather production) reduce the scope for differences among lineages.
(4) *What ecological and physiological factors drive variation among birds in elemental composition? *We predicted that variation within and among species should reflect differences in body size arising from allometric scaling of skeletal investment [12,13], as well as seasonal fluctuations in fat reserves driven by migration and overwintering [26,27], rather than differences in elemental intake associated with diet.

## 2. Results and discussion

### (a) Distinctive stoichiometry of different bird tissues

**Figure 1.**
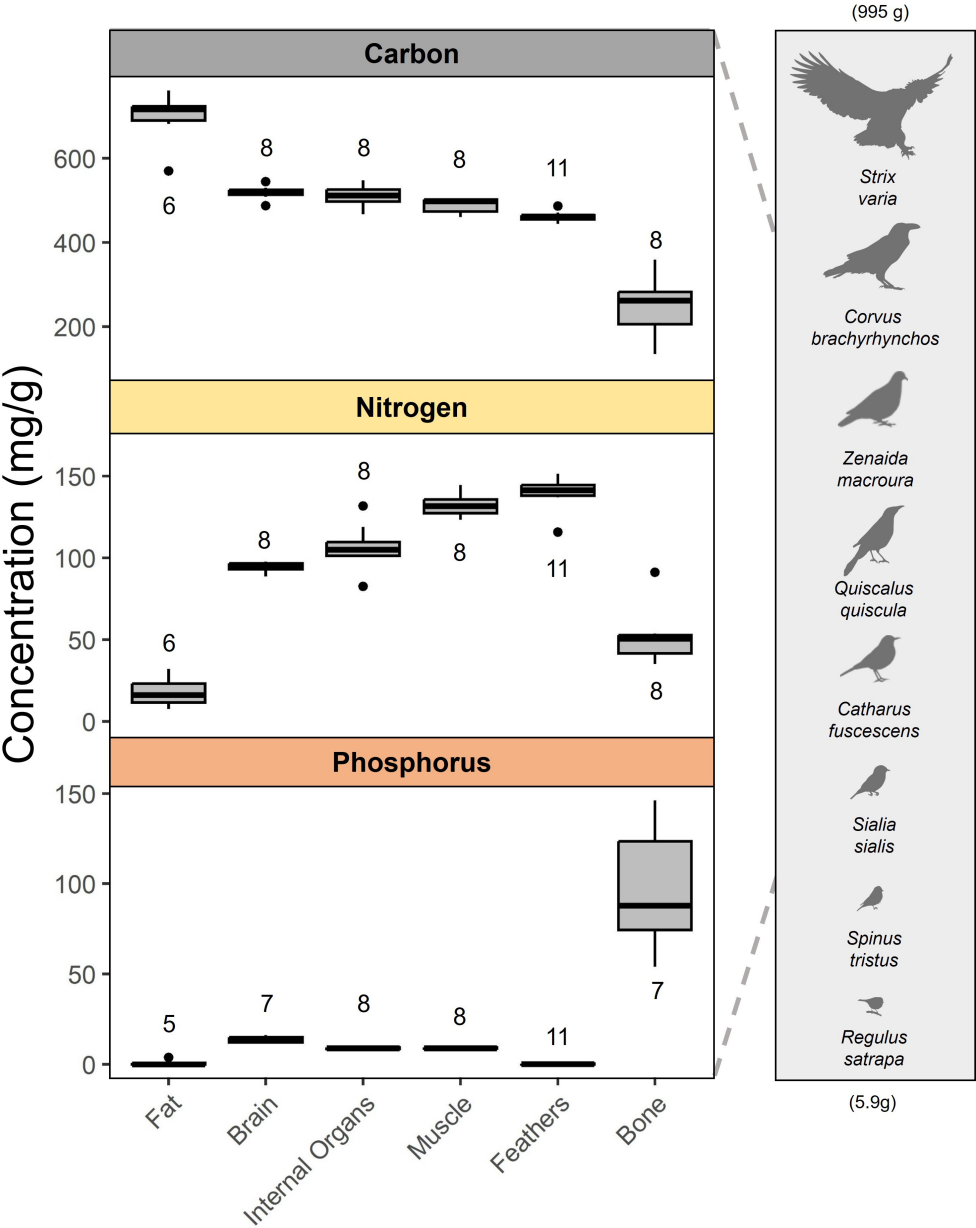
Concentrations of carbon (C), nitrogen (N) and phosphorus (P) in tissues of select birds, shown on the right side of the figure. Each tissue type exhibited a distinctive elemental composition, though brains and internal organs had similar %C, and internal organs and muscle were not significantly different in %C and %P (electronic supplementary material, table S3). Numbers adjacent to boxes represent sample sizes for each tissue. Body and wing feathers were processed and analysed separately for *Strix varia*, *Corvus brachyrhynchos* and *Regulus satrapa* (largest and smallest sampled species). In some birds, insufficient bone, brain or fat tissue prevented nutrient analysis (electronic supplementary material, table S2).

We observed significant variation in the elemental composition of different tissue types (figure 1; Kruskal–Wallis, *p* < 0.001 for %C, %N and %P), as predicted based on biochemical properties. For the subset of species that we dissected for tissue analyses (*n* = 8 species, though see tissue-specific sample sizes on figure 1 and electronic supplementary material, table S2), pairwise comparisons confirmed that fat had significantly higher %C and lower %N (*p* < 0.001) than all other tissue types and lower %P than all tissues (*p* < 0.01) except feathers (electronic supplementary material, table S3). In fact, every tissue type showed a distinctive composition, though brains and internal organs did not differ in %C, while internal organs and muscle were similar in both %C and %P (electronic supplementary material, table S3).

As expected [1,21], bone exhibited 6- to 605-fold more P (mean: 9.7% ± 3.4 SD) than other tissues, reflecting the prevalence (60−70% by mass) of calcium-phosphate bone mineral (hydroxyapatite) for skeletal structure [28]. Conversely, fat contained very little P (0.09% ± 0.16 SD) but the highest C concentration (69.5% ± 6.6 SD), reflecting the C-rich chemistry of triglycerides (fatty acids) for energy storage [29]. In contrast to the low N content of bone (5.2% ± 1.6 SD) and fat (1.8% ± 1.0 SD), feather and muscle were N-rich (14.0% ± 1.0 SD and 13.2% ± 0.7 SD, respectively) due to their prevalence of N-rich proteins [30]. However, unlike muscle, feathers contained virtually no P (0.02% ± 0.02 SD) since β-keratin, the main constituent of feathers, is a non-living tissue that does not require P for cell structure or maintenance [30]. These results confirm that (i) tissue stoichiometry reflects the chemistry of the dominant molecules [1] and that (ii) significant stoichiometric differences among tissue types can drive variation in whole-body stoichiometry within and across taxa through alternative tissue investment strategies.

While stoichiometric variation within individual tissues was generally low (figure 1), Brown–Forsythe tests revealed significant heterogeneity in variance for %C and %P among tissues (*p* = 0.017 and *p* < 0.001, respectively), whereas %N variances were broadly similar (*p* = 0.40). This pattern is primarily driven by bone, which varied twofold in %C, %N and %P (figure 1). Differences in bone mineral chemistry do not appear to explain bone’s stoichiometric variability, as subsequent analyses confirmed that Ca:P ratios were comparatively invariant (2.13 ± 0.11 SD; *n* = 7). Rather, we attribute this variation to differences in bone mineral density: the relative investment in mineral (P-rich hydroxyapatite) versus marrow (e.g. C- and N-rich collagen). May & El-Sabaawi [21] found that raptors, such as the barred owl (*Strix varia*) and Cooper’s hawk (*Accipiter cooperii*) among our sampled species, feature more than twice as much mineral content in their bones as songbirds—probably an adaptation to strengthen limbs for capturing prey. Indeed, C:P was lowest in the largest carnivorous species we sampled, yet variability among smaller frugivorous and granivorous species obscured any significant relationships with diet or body size (electronic supplementary material, figure S2). These intriguing results suggest that further study of skeletal investment strategies, including distinctions between bone types and mineral versus marrow content, may yield additional stoichiometric insights.

### (b) Birds exhibit distinct elemental composition among vertebrates

**Figure 2.**
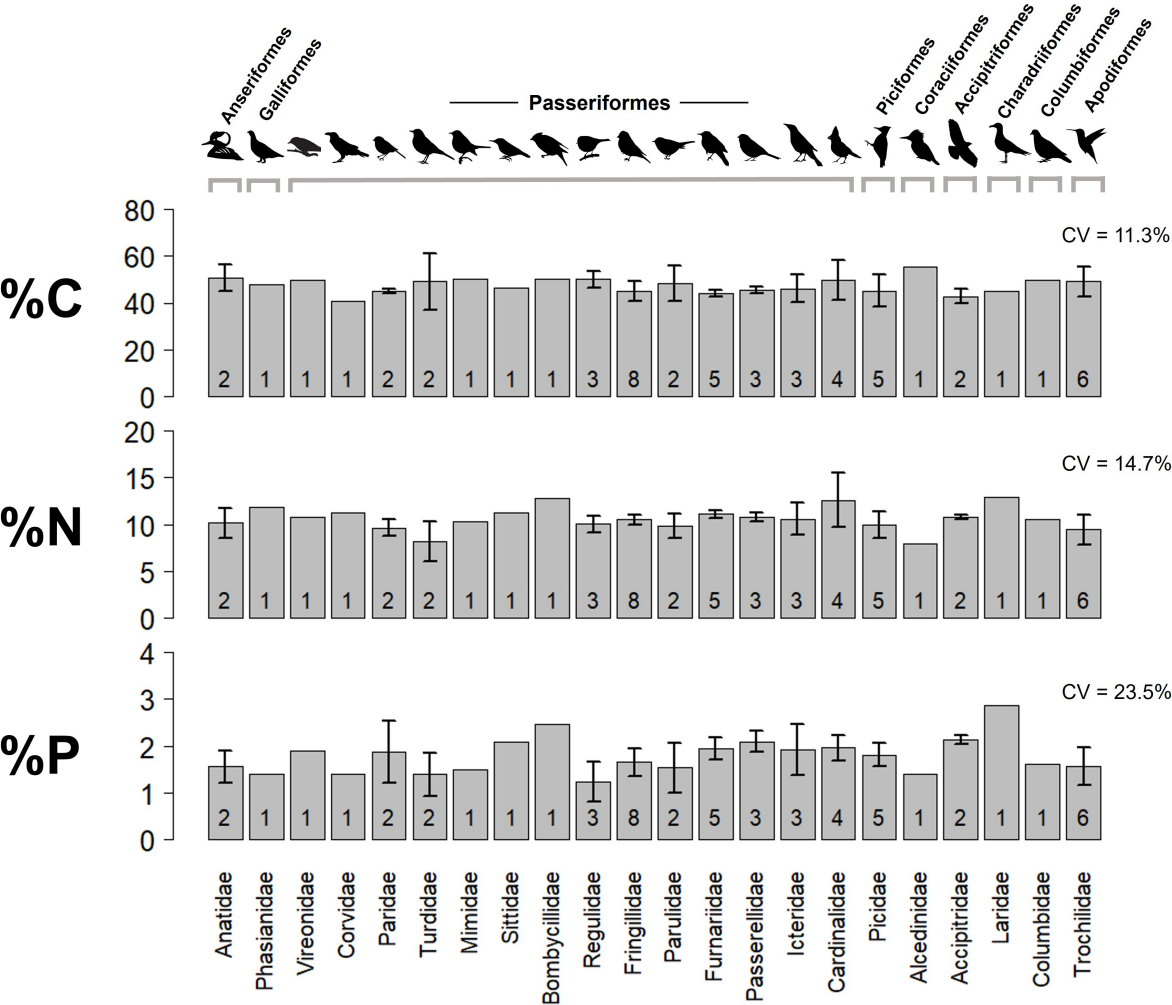
Variation in the carbon (%C), nitrogen (%N) and phosphorus (%P) content of birds, with taxa ordered phylogenetically by family (bottom) and order (top). No significant differences between species, family or order were detected, and phylogenetic signal was also weak and non-significant. Error bars represent SD and numbers on bars represent sample sizes per family. Note: the order *Passeriformes* represents 60% of all birds worldwide.

**Figure 3.**
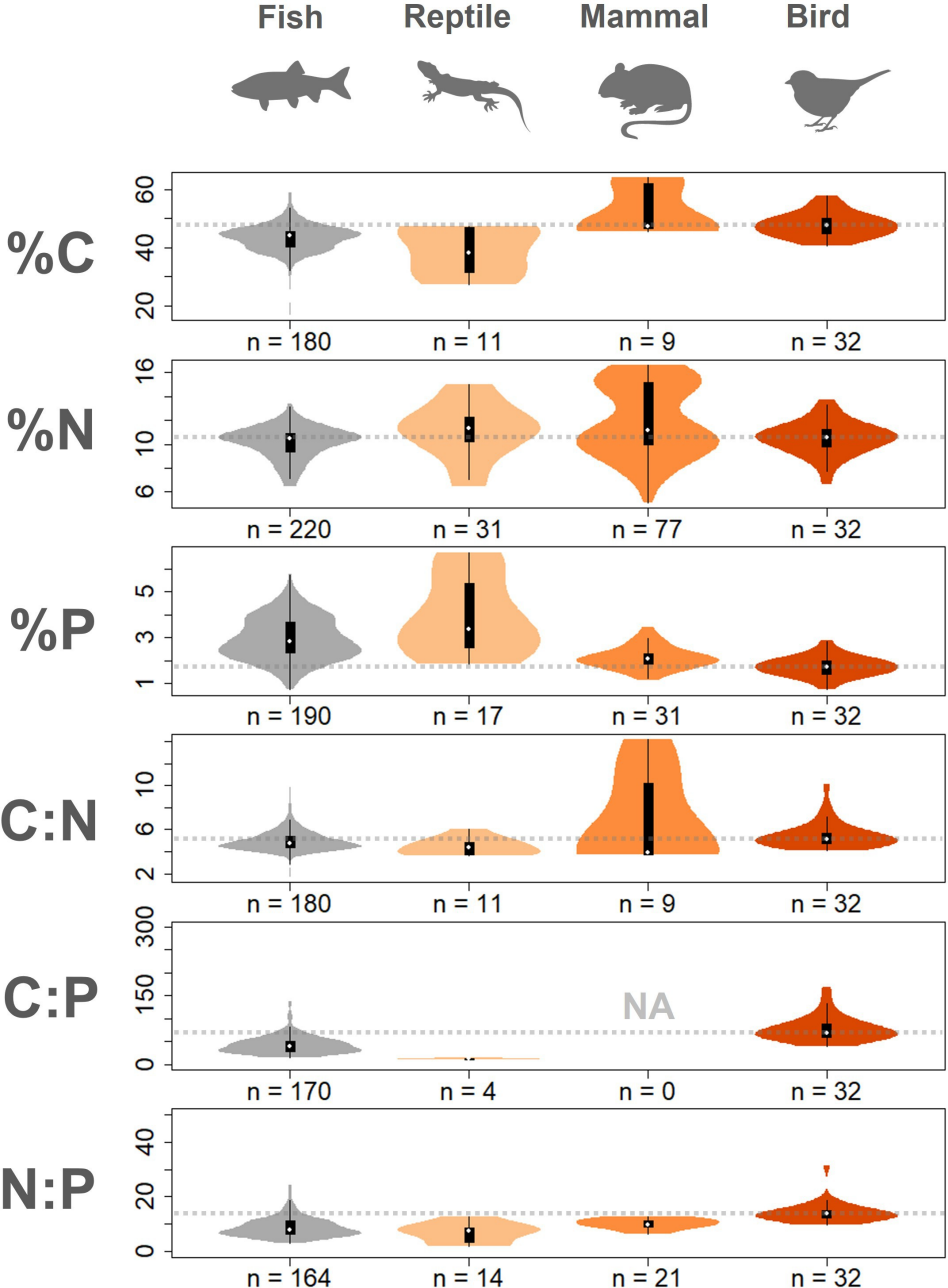
Comparison of the body stoichiometry of birds in this study to other vertebrate groups (non-bird data from Andrieux et al. [8]). Birds display significantly lower phosphorus content (%P) and higher C:P and N:P ratios compared to other vertebrate groups (electronic supplementary material, table S4). Birds also exhibit the highest carbon (%C) content, though comparable to limited data available for mammals. Violin plots depict the distribution and density of data for each vertebrate group, with white dots indicating the median value. *n* denotes the number of species represented in each violin plots. Dotted horizontal lines denote the median values specifically for birds (to facilitate comparisons).

We observed substantial variation among birds in body elemental content, including C (33.8−61.5%), N (6.7−16.9%) and P (0.73−2.9%), as well as molar ratios (C:N of 4.1−10.1; C:P of 40.5−168.5; N:P of 9.7−31.2) (figure 2). On average, birds exhibited significantly lower %P (median = 1.7% ± 0.4 SD; *n* = 32 species) and significantly higher C:P and N:P ratios compared to other vertebrates (figure 3; electronic supplementary material, table S4). Fishes, for example, had a median %P that was ~65% higher than birds (2.8% ± 0.9 SD; *n* = 190 species), while mammals were only moderately higher (2.1% ± 0.5 SD; *n* = 31 species). Brown–Forsythe tests also indicated that birds had significantly lower variability in %P than fishes and reptiles (*p* < 0.001) but were similar to mammals (electronic supplementary material, table S5).

These patterns align with our prediction that anatomical adaptations for flight may constrain whole-body P content and limit interspecific variability. Unlike fishes, whose lack of gravitational constraints in water allows them to adjust investments in skeletal structures (e.g. dermal armour) without altering their mobility or durability [4], birds are under selective pressure to minimize skeletal mass and volume to meet the high energetic demands of flight [31]. While flightless (<1% of species) and diving birds generally display thicker-walled bones and bone-mass redistribution (e.g. wings to legs [32]), these differences have minor effect on overall skeletal mass compared to differences among vertebrate taxa [33]. Mammals and reptiles also exhibit associations between skeletal mass and terrestrial locomotion on land [32]; however, this has not precluded the evolution of extreme skeletal adaptations, including bony defensive armour (e.g. shells) and weaponry (e.g. antlers and tusks), with profound impacts on body P [11,21]. Even in bird species with larger bills (e.g. toucans) or casques (e.g. hornbills), the keratinous composition of these external structures keeps them lightweight and minimizes P investment [34].

Prange et al. [12] previously noted that, despite having exceptionally pneumatic (air-filled) bones, bird skeletons are not substantially lighter than those of mammals with similar body mass, contradicting conventional wisdom. Dumont et al. [31] further addressed this apparent contradiction by showing that, although birds have thinner bones, they compensate by increasing bone mineral density—an adaptation for flight that maintains structural strength while reducing bone volume (also observed in bats [31]). Indeed, despite the strong selective pressure to reduce skeletal mass and conserve energy during flight, birds also experience significant mechanical forces (e.g. flight generation, impact upon landing) that require extreme skeletal strength [21]. While we found that %P in birds was more similar to that of mammals than to other vertebrate groups—supporting the idea that birds’ seemingly lightweight skeletons may be offset by increased bone mineral density—the finding that birds are significantly lower in %P than mammals nevertheless complicates the notion that skeletal mass alone explains birds’ lower P content. Instead, we infer that birds limit their total body P through another flight-related mechanism: allocating nearly one-fifth of their body mass (19.5% ± 6.1 SD; *n* = 56) to feathers, which contain negligible P (figure 1). The combination of dense bones and extensive investment in P-poor integument probably underlies the generally lower and less variable P content in birds.

Interestingly, despite their substantial investment in N-rich, keratinous feathers, birds exhibited %N values that were statistically similar to those of other vertebrates (figure 3; electronic supplementary material, table S4) and showed comparable levels of variation (electronic supplementary material, table S5). Across vertebrates, muscle tissue represents the dominant N reservoir and is presumed to vary less across vertebrate body plans than other tissue types [35]. Beyond feathers, other keratinous structures may impact vertebrate N content: in mammals, dense fur may comprise a notable portion of body mass for particular species (though generally low [35]), whereas reptilian scales probably contribute minimally. It is possible that the high N content of feathers in birds may be balanced by investment in tissues with lower N concentrations, such as fat and bone (figure 1). While bone is an unlikely candidate given birds’ restricted skeletal mass, birds may, on average, maintain relatively large fat stores, consistent with their status as highly metabolic endotherms [36].

In line with these ideas, birds exhibited significantly higher %C and C:N ratios than ectothermic reptiles (median %C = 25.1% higher) and fishes (8.3% higher), while more closely resembling endothermic mammals (figure 3; electronic supplementary material, table S4). Birds in our dataset exhibited a wide range of fat content, with many individuals showing visible fat deposits around their furcular and abdominal region. Endothermy may necessitate larger stores of C-rich lipids to buffer against variable food supply in the face of high metabolic demands [29,36], especially in colder regions or seasons. However, many ectotherms also store fat for insulation and excess energy during brumation [37]. For example, fishes can vary in lipid content based on temperature and life-history stage (e.g. migration), contributing to body stoichiometry differences across species [38]. Accounting for variation in body C content arising from lipid storage is essential for clarifying the role of thermoregulation, migration, reproduction and other seasonal needs on vertebrate body stoichiometry. Our results suggest that additional intraspecific and interspecific comparisons among birds, along with cross-vertebrate group comparisons within a single study, would greatly advance this goal.

### (c) Evolutionary identity poorly predicts bird elemental composition

Despite considerable variation among birds, taxonomic and phylogenetic affinities were poor predictors of their body stoichiometry. Phylogenetic signal was weak and non-significant (λ < 0.01; *p* > 0.05; electronic supplementary material, figure S1), reflecting the considerable compositional variation among individuals within species (electronic supplementary material, figure S4). This intraspecific variation does not appear attributable to analytical error, as our replicate analyses of individual birds and quality control tests indicate high precision (electronic supplementary material, text S1).

Multivariate analysis of variance (MANOVA) tests showed no significant species- or order-level differences in elemental content (%C, %N, %P) or stoichiometric ratios (C:N, C:P, N:P) (electronic supplementary material, table S6), but family-level affiliation had a significant effect on combined elemental percentages (Pillai’s trace = 0.881, *p* = 0.042). However, follow-up univariate tests and pairwise comparisons with Holm correction found no significant differences among families for C, N or P, suggesting that any differences in elemental composition among bird families are subtle or at least inconsistent.

The lack of detectable phylogenetic influence on bird stoichiometry in our dataset should not be interpreted as evidence that between-group variance in body composition does not exist. Though constraints associated with flight may limit the scope for stoichiometric differentiation in birds, there remains notable variation within species, families and orders that indicates a diversity of feasible body stoichiometries. Small and unbalanced sample sizes for some groups in our survey could have obscured taxonomic or phylogenetic patterns, as could heterogeneity within groups in ecological factors.

### (d) Ecological and physiological influences on bird stoichiometry

**Figure 4.**
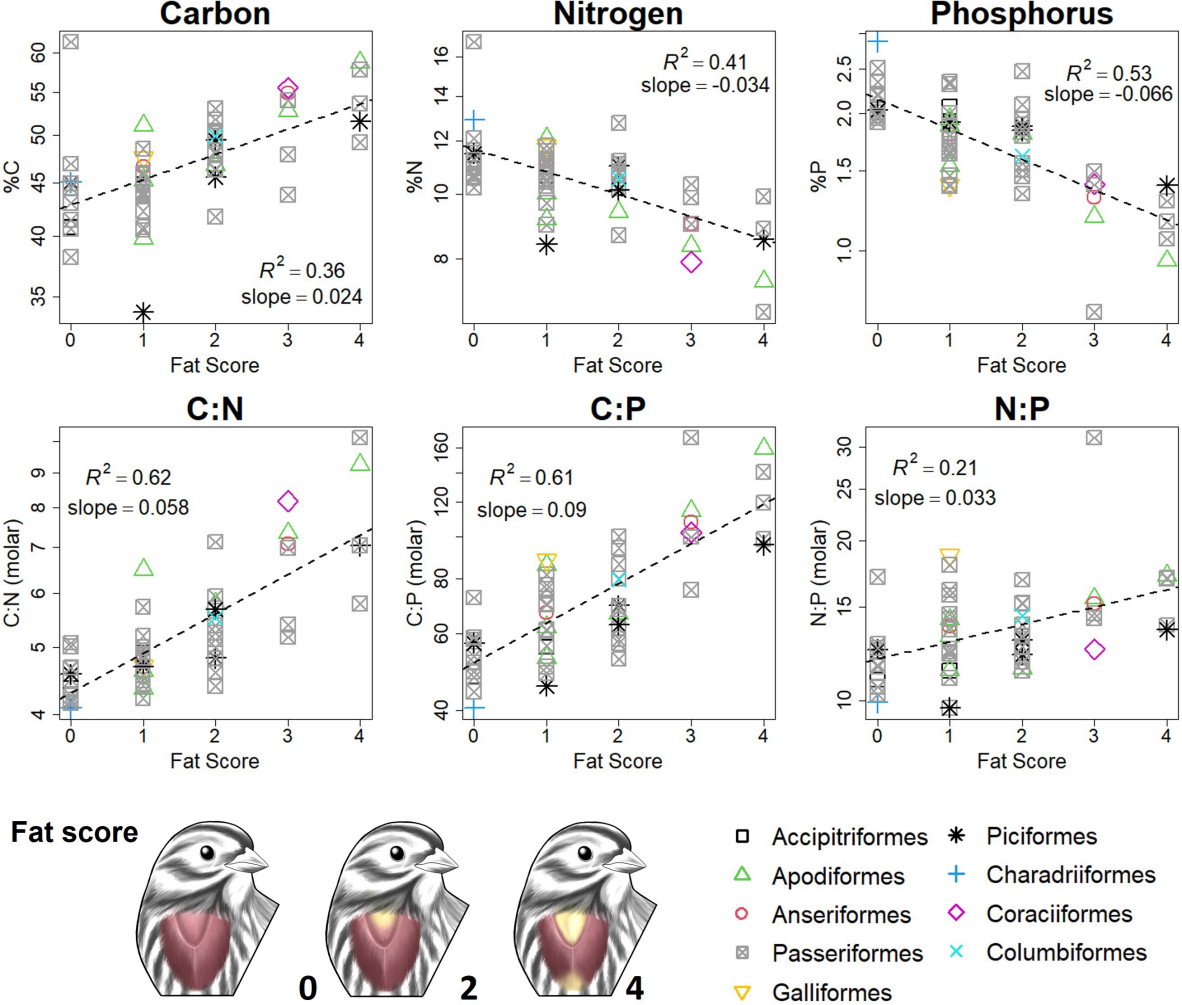
Influence of fat storage on the elemental composition of birds (all relationships shown are statistically significant: *p* < 0.001). Fattiness was the primary axis of compositional variation among birds due to its extreme stoichiometric signature (figure 1), substantial contribution to body mass and well-documented seasonal variability [26,27]. Fat production leads to greater carbon (C) content in birds while reducing the proportions of nitrogen (N) and phosphorus (P). Nitrogen also increases relative to phosphorus during fatty periods, potentially reflecting a compensatory increase in muscle mass. Each point represents an individual bird (*n* = 56). Fattiness was assessed using standard ordinal fat-scoring methods based on visible subcutaneous fat following Helms & Drury [39].

We found that, among our measured traits, fattiness was the single best predictor of bird body stoichiometry both within and across species (table 1). Fattiness was positively associated with C content and negatively related to P and N contents (figure 4). Fat storage is a critical strategy for birds to meet energy demands of migrating [26], overwintering [27] and parental care [40]. Birds can more than double their body weight during fat accumulation periods [26,27], as was evident from our measurements (e.g. ruby-throated hummingbird: 0.86−1.9 g dry weight; electronic supplementary material, figure S4). Accordingly, qualitative fat scores explained 36−53% of variation in elemental content and 21−62% of variation in elemental ratios (table 1). N:P ratios also increased with fat scores (figure 4), possibly reflecting the need to boost N-rich muscle mass to support flight while laden with fat, whereas skeletal P remains fixed [26,41].

**Table 1.** Best performing regression models for predicting the body stoichiometry of birds (*n* = 56) based on AIC model selection. Models with ΔAIC = 0 (best fit) are bolded for each stoichiometric variable. Fat storage was the best single predictor of stoichiometric variables, while feather content explained additional variation for stoichiometric variables involving nitrogen (N). Body mass, diet, age class and sex were not included in any top models.

		%C				%N				%P			
model	*K*	*ΔAIC*	*AICcWt*	*R^2^*	*p*	*ΔAIC*	*AICcWt*	*R^2^*	*p*	*ΔAIC*	*AICcWt*	*R* ^2^	*p*
*fat*	3	**0.00**	0.44	0.36	<0.001	16.37	0.00	0.41	<0.001	**0.00**	0.52	0.53	<0.001
*mass + fat*	4	1.22	0.24	0.37	<0.001	18.24	0.00	0.41	<0.001	2.19	0.18	0.53	<0.001
*fat + feather*	4	2.13	0.15	0.36	<0.001	**0.00**	0.48	0.58	<0.001	2.27	0.17	0.53	<0.001
*mass + fat + feather*	5	3.17	0.09	0.37	<0.001	2.37	0.15	0.58	<0.001	4.58	0.05	0.53	<0.001
*fat + feather + carnivory*	5	4.25	0.03	0.36	<0.001	0.96	0.29	0.59	<0.001	4.33	0.06	0.53	<0.001

		C:N				C:P				N:P			
model	*K*	ΔAIC	AICcWt	*R^2^*	*p*	ΔAIC	AICcWt	*R^2^*	*p*	ΔAIC	AICcWt	*R^2^*	*p*
*fat*	3	14.93	0.00	0.62	<0.001	**0.00**	0.52	0.61	<0.001	3.28	0.08	0.21	<0.001
*mass + fat*	4	17.23	0.00	0.62	<0.001	2.30	0.16	0.61	<0.001	5.60	0.02	0.21	<0.01
*fat + feather*	4	**0.00**	0.47	0.72	<0.001	2.16	0.18	0.61	<0.001	**0.00**	0.40	0.29	<0.001
*mass + fat +* *feather*	5	1.07	0.27	0.73	<0.001	4.52	0.05	0.61	<0.001	2.21	0.13	0.29	<0.001
*fat + feather* *+ carnivory*	5	1.95	0.18	0.72	<0.001	4.06	0.07	0.62	<0.001	0.70	0.28	0.31	<0.001

Interestingly, the higher relative variability in %P (CV = 23.5%) compared to %C (11.3%) and %N (14.7%) in birds (figure 2) may be caused by fat (C-rich) and muscle (N-rich) tissue masses rising and falling together; these large, coupled shifts will dramatically change whole body %P even though the absolute amount of skeletal bone is both small and invariant. Thus, we conclude that the well-documented seasonal dynamics of fat reserves have previously unrecognized consequences for bird body stoichiometry, and our inadvertent sampling across these swings could have obscured any taxonomic or phylogenetic signals in body composition (figure 2).

After accounting for the effects of fat, feather mass was also an important predictor of the elemental composition of birds (table 1). Feathers—the key evolutionary innovation that enables birds to fly—constituted an average of 19.5% ± 6.1 (SD) of total dry body weight (range: 7.7−36.2%). Their substantial mass and N-richness of keratin (figure 1) make feathers an important compartment of N in birds, accounting for a mean 24.9% ± 12.7 (SD) of total body N. Accordingly, feather mass explained an additional 17, 10 and 8% of variation in the best performing multiple regression models (ΔAIC = 0) for bird %N, C:N and N:P, respectively (table 1). Some variation in feather mass may stem from body size and taxonomic differences. For example, hummingbirds (Apodiformes), the smallest-bodied bird order, featured the lowest relative feather mass, providing an anchor point for the general increase in feather mass with body size (electronic supplementary material, figure S5). Feather mass can also vary by age, season and life history [42,43], though these factors were not evident in our data. Nonetheless, the variation in feather mass among our sampled species played a smaller role in shaping bird body %N and stoichiometry than did fat storage.

Given their high N content, the shedding and replacement of feathers during moulting has profound implications for nutrient allocation and demand. Most birds moult annually or bi-annually [44], but the frequency and extent vary by migration strategy [45], age [43] and environment [46]. For example, tropical albatrosses (*Diomedeidae spp*.) moult twice as fast as sub-Antarctic species, yet this process can occur over several years [45]. In contrast, many waterfowl (Anseriformes) moult synchronously twice a year, regrowing their feathers over several weeks [45]. Our measurements suggest that fully moulting requires replacing ~25% of body N, with no mechanism for recouping lost N from shed feathers. This facet of bird ecology represents one of the most extreme cyclical shifts in body chemistry among animals; shedding dermis (squamates) and fur/hair (mammals) are comparatively trivial degrees of tissue renewal. Since moulting ensures that birds are able to complete long migrations between breeding and overwintering grounds, the stoichiometric dynamism of birds arising from moulting should be seen as a major secondary consequence of avian flight evolution. Nonetheless, these seasonal changes are easy to overlook in static snapshots of body stoichiometry. Moreover, we might predict that the pressing need for N during plumage renewal would strongly favour consumption of animal prey rather than plant material, thereby altering species ecology and interaction webs well beyond birds themselves.

In contrast to our expectations, body size was not a strong predictor of bird body stoichiometry. Positive allometric scaling of skeletal mass has been presumed to influence P storage and transport by mammals [20,47]. However, we found isometric size-scaling of P content for birds (0.016 × BM^1.02 (CI: 0.98–1.07)^; electronic supplementary material, figure S3) rather than the shallow positive skeletal scaling reported previously (BM^1.07–1.08^) [12,13]. This result may reflect a weak allometric increase in feather mass containing virtually no P (0.16 × BM^1.05 (CI: 0.99–1.11)^; electronic supplementary material, figure S5), dampening total P increases from skeletal mass allometry. While smaller birds exhibit higher growth and metabolic rates, potentially requiring more body P for RNA synthesis and ATP production [1,2], we found very stable and low P content in non-bone tissues (figure 3), suggesting minimal influence on whole-body P. Nonetheless, birds may exhibit distinct stoichiometries through ontogeny linked to the timing and rates of bone and muscle development [14,48]. Such variation warrants further exploration in birds, whose rapid growth until fledging and diverse parental investment strategies (e.g. altricial versus precocial) surely make them unusually vulnerable to nutrient limitation.

Although ecological stoichiometry theory predicts that differences in access to nutrients arising from contrasting diets could give rise to divergent body composition patterns, we found no evidence that wide variation in trophic ecology affects avian body stoichiometry (table 1). This lack of alignment is consistent with other vertebrate studies [8,49,50]. While carnivory appeared in the best model for explaining body N:P in birds (table 1), its coefficient was not significant (*p* > 0.05). Interestingly, birds that eat other vertebrates did exhibit slightly higher (but not significant) body %P and lower C:P and N:P content (electronic supplementary material, figure S8). This pattern resembles observations in carnivorous fishes [50], but that could be due to correlation with other traits that enhance predator performance (e.g. denser bones needed to attack and subdue prey, or larger body size [21]). Some birds show flexibility in skeletal storage of P and Ca wherein medullary bone can be demineralized to support reproductive needs [21,28], so it remains plausible that higher P intake from consuming vertebrates could enable bone fortification. Nevertheless, our results suggest that diet has a minimal influence on bird stoichiometry compared to overall constraints imposed by flight adaptations and seasonally fluctuating tissue composition.

The apparent non-effect of diet on bird body stoichiometry carries important implications for avian biology and ecology. Maintaining a relatively consistent stoichiometry across species with vastly different dietary access to elements [51] suggests that birds—both species and individuals—encounter a wide range of stoichiometric imbalances. Such imbalances at the individual scale can theoretically influence nutrient intake, storage and behaviour, ultimately driving population-level effects on nutrient cycling and other ecological functions [52]. Birds adapt to nutrient limitation by foraging across large areas, whereby flight enables them to seek nutrient-rich foods in heterogeneous environments. For example, many birds shift their diet towards Ca-rich foods before egg-laying [53] and C-rich foods before migration for energy storage [27]. Thus, the mobility granted by flight helps birds maintain stable body stoichiometry and high metabolic rates amid time-varying nutritional demands, while also enhancing their functional roles in ecosystems (e.g. nutrient and seed transport [15,16,18,54]). While some birds adjust moult timing, delay migration or reduce growth when faced with nutritional limitations [45], such plasticity appears less common in avian ecology than for other vertebrates [2,55].

### (e) Overall implications of bird stoichiometry for zoogeochemistry

Birds can have profound ecological impacts by recycling and transporting nutrients in their waste [15–18]. Seabirds, for example, are estimated to transport ~591 Gg N y^−1^ and ~99 Gg P y^−1^ from sea to land annually [16], and freshwater birds can contribute >75% of total P inputs into lakes seasonally or annually [15,56]—capable of fuelling primary productivity and shaping community structure [57]. Among vertebrates, birds possess exceptionally high mobility and metabolism [58], releasing excreted (metabolic by-products) and egested (undigested) waste simultaneously through their cloaca, contributing to their unique zoogeochemical roles. Ecological stoichiometry theory estimates an organism’s nutrient recycling as the imbalance between dietary nutrient intake and nutrient retention to grow body tissues [1]. Since we find that the bulk elemental composition of birds is relatively consistent, wide variation in diet stoichiometry must produce commensurate variation in nutrient release. This logic accords with studies of avian nutrient recycling; birds release more waste nutrients when their diet is either stably higher in nutrients (e.g. carnivores versus herbivores) or shifts towards nutrient-rich foods [15,16]. However, nutrient recycling should also be affected by body stoichiometry, such as the differences in fat, feather and muscle investment documented in our survey of birds, which may secondarily influence nutrient release. For example, migratory birds may sequester more C and release wastes with lower %C, C:N and C:P in order to store fat before migration [26,27]. More research is needed to evaluate how fat storage and moulting influence nutrient release by birds, and whether concurrent dietary shifts help to meet shifting needs for assimilation during these periods.

Beyond their wastes, animals can affect ecosystems by transporting nutrients stored in their bodies, whereby mass movements create major inputs or removals of key nutrients [24]. Baleen whales, for example, convey an estimated 3784 T of N to their wintering grounds annually via mass migration in the form of carcasses, placenta and urea waste. Among animals, birds are renowned for migrating great distances en masse [23]. Their relatively consistent elemental composition suggests that factors such as migrant biomass, movement corridors and mortality rates are the key determinants of the size and nature of potential nutrient subsidies from birds. To date, bird migrations have rarely been considered as nutrient vectors [22], in part due to lack of body composition data [8,19]. However, several aspects of bird biology and ecology, including their rapid growth, high metabolic rates, global distribution, high mobility and demographic patterns (e.g. elevated mortality during migration) suggest they have high potential to deliver important subsidies to many different types of ecosystems worldwide.

### (f) Study limitations and future research directions

Our bird body stoichiometry results set the stage for new insights and perspectives in avian biology and beyond, yet several limitations of our study also point to exciting opportunities for future research. First, we prioritized phylogenetic coverage to capture a wider range of bird lineages and, by extension, greater trait diversity, rather than achieving extensive within-species replication. Limited within-species replication (*n* = 1−6) surely reduced our capacity to represent typical values for each species, thereby weakening our ability to detect phylogenetic and taxonomic influences. However, our evidence that fattiness and feathers give rise to substantial compositional variation within species also provides a cautionary perspective, since most body composition surveys presume that a snapshot approach is sufficient to quantify differences among species [4,10,50]. Thus, it would be best to not only increase within-species replication in order to assess cross-species patterns but also to survey the same species across seasons or ontogeny to further elucidate intraspecific influences on bird body stoichiometry.

Second, while we included a wide range of key avian traits from birds in North America (many which are neotropical migrants), the sheer diversity of bird species—including extreme cases of evolved diets (e.g. leaf-eating: hoatzin, *Opisthocomus hoazin*), body size (e.g. >100 kg: ostrich, *Struthio camelus*), life history (e.g. ice-dwelling and deep-diving: emperor penguin, *Aptenodytes forsteri*) and body composition (e.g. exceptionally thick-casqued: helmeted hornbill, *Rhinoplax vigil*)—may present unique exceptions to the patterns discussed here. Expanding coverage of bird biodiversity will enable more robust generalizations about avian body stoichiometry. For instance, our samples were limited to North American birds, yet year-round tropical species may exhibit distinct stoichiometries and nutrient demands associated with reduced fat and muscle composition and longer inter-moult periods [46,59]. Importantly, we leveraged access to recently deceased birds donated to a museum from the public, and such opportunities abound worldwide for obtaining diverse specimens for non-invasive stoichiometry research.

Third, our analysis was limited to C, N and P in birds, yet stoichiometric research can be greatly enriched by examining broader elemental profiles or ionomes [60]. Many other elements play foundational roles in organismal biology and ecology [61], influence bird fitness and survival [53] and may exhibit body stoichiometry patterns distinct from those reported herein due to their unique associations with different tissues. For example, ionomic analyses of fish species have revealed substantial elemental variation across tissues for 14+elements, demonstrating that elemental profiles clustered more strongly by tissue type than body size, sex or species [60]. While we found impressive stoichiometric differences among tissues with a limited menu of elements and specimens (figure 3), we may only be scratching the surface of compositional variation and its ecological consequences. Comprehensive multi-elemental measurements of both individual tissues and whole bodies will surely raise new questions and perspectives in stoichiometric research with implications well beyond birds.

Fourth, quantifying the elemental composition of birds enhances understanding of their requirements for growth and production of specialized tissues. Yet, there remains ample opportunity to integrate stoichiometric data with physiological information, such as the metabolic costs and maintenance requirements of tissues, to elucidate nutritional and energetic constraints on bird biology and ecology. For example, integrating the additional N required for moulting into a stoichiometric understanding of bird ecology could be achieved using an approach akin to threshold elemental ratio modelling [3] wherein C respired during metabolism is accounted for beyond predictions from body composition alone. Such explorations are particularly relevant in the context of global climate and habitat changes, which are altering many aspects of bird physiology, phenology and nutrient intake [62].

Finally, our findings present exciting new opportunities to quantify bird contributions to elemental cycling. Combining body stoichiometry profiles with extensive avian trait data may enable powerful predictions of the magnitude and spatiotemporal dimensions of nutrient recycling by birds. These predictions can be empirically tested and incorporated into spatially explicit zoogeochemical models to illuminate the holistic contributions of bird communities to ecosystem functioning and global nutrient cycles [52,63]. It is particularly exciting to consider how bird body stoichiometry data like ours could be combined with increasingly robust avian abundance and distribution data [23,25] to quantify nutrient transport in bird bodies at broad scales. These explorations can include the world’s great annual bird migrations (e.g. >8 billion birds in North America [23]), as well as historic movements, such as the billions of now-extinct passenger pigeons (*Ectopistes migratorius*) which once filled the skies of eastern North America. Better integrating birds into the zoogeochemical research agenda will advance fundamental understanding of ecological stoichiometry, provide new avenues for public engagement, help conservation scientists to restore ecosystems [63], enhance environmental resilience [17] and achieve bird conservation [25] around the world.

## 3. Methods

### (a) Laboratory analysis methods

We procured fully intact bird specimens that had been scavenged and donated by the public to the Cornell Museum of Vertebrates between 2006 and 2022. The most common cause of mortality for scavenged birds was window or vehicle strike, though many of the causes were reported as unknown (electronic supplementary material, table S1). All specimens were sourced from central New York State, USA, and were immediately frozen and held at −18°C upon collection. We excluded birds that showed any signs of degraded body condition such as rotting, parasites or extreme emaciation.

Before processing birds for analysis, we identified, sexed, aged and weighed individuals. We then defeathered each bird and assessed fat content using standard ordinal fat-scoring methods based on visible subcutaneous fat (see table 4 in Helms & Drury [39] for fat-scoring protocol) with a minimum score of zero (no fat visible) and a maximum score of five (furcular region and lower abdomen filled with fat). Individual birds were then dissected to remove all dietary material from the crop and gizzard, and their bodies and feathers were freeze-dried at −40°C until a consistent weight was recorded (typically one to two weeks). After weighing, we homogenized full bodies to a fine powder using a combination of a spice grinder (VEVOR 2500g) and mortar and pestle with liquid nitrogen. For larger birds, we separated the body into major segments (e.g. legs, wings, torso, head), coarsely divided each segment using a meat grinder (Avantco MG12R), ground each segment individually into a fine powder (using methods above) and then pooled the powders to create a fully homogenized sample. For obtaining individual tissue measurements, we used separate full-body individuals and similarly identified, sexed, aged and weighed each before dissection (figure 1). Dissection was performed to sub-sample fat, bone, muscle, wing feathers and body feathers from the bird, whereas the entirety of the brain and internal organs were sampled and homogenized. We fully freeze-dried target tissues and ground them into a fine powder using mortar and pestle and liquid nitrogen.

Homogenized powder samples were sub-sampled and analysed for C and N content using a Carlo Erba NC2500 elemental analyser (EA). We measured P and Ca content in digested samples using inductively coupled plasma-optical emission spectrometry (ICP-OES). Samples were digested using a mixture of 70% HNO_3_ and 30% H_2_O_2_ at 180°C in a Milestone Ethos microwave digestor. We used in-house standards for calibrating EA and ICP-OES analyses. We validated our EA measurements using certified international reference materials provided by the International Atomic Energy Association and validated ICP-OES analyses using the NIST1570A standard from the National Institute of Standards and Technology (electronic supplementary material, text S1).

### (b) Statistical analysis methods

We calculated body elemental composition using the following equation:


(3.1) $$Composition_{X_{i}}=\left(\left(BM_{i}*B_{conc_{X_{i}}}\right)+\left(FM_{i}*F_{conc_{X}}\right)\right)÷\left(BM_{i}+\;FM_{i}\right),$$

where ${Composition}_{X_{i}}$ is the concentration of element X in species i (in mg g^−1^ dry weight (DW)), ${BM}_{i}$ is the defeathered body mass of individual i (in g DW), $B_{{conc}_{X_{i}}}$is the concentration of element X in the defeathered and dissected body measurement of individual i (in mg g^−1^ DW), ${FM}_{i}$ is the feather mass of individual i (in g DW) and $F_{{conc}_{X}}$ is the mean concentration of element X in feather tissue samples. We assumed a standard elemental concentration across feather tissues based on the minimal variation observed for feather sub-samples (figure 1). For all analyses, we used the combined body and feather mass in DW to eliminate potential variation in water content induced by differences in mortality or freezer time. Birds were on average 59% water by weight, and we present the conversion factor from DW to fresh weight (FW) in electronic supplementary material, table S1. These and all subsequent analyses were conducted using base R v. 4.0.3 [64].

(1) *How distinct are the elemental compositions of different bird tissues? *We assessed differences in %C, %N and %P among tissue types using non-parametric methods due to non-normal distributions and heteroscedasticity in the data (see figure 1 and electronic supplementary material, table S3 for sample sizes). Kruskal–Wallis tests were applied to detect overall differences in medians across tissues, followed by pairwise Wilcoxon rank-sum tests with Benjamini–Hochberg correction to identify specific group differences. We evaluated variance equality using Brown–Forsythe tests.
(2) *How does the elemental composition of birds compare to other vertebrates?* We examined statistically significant differences in %C, %N and %P, as well as C:N, C:P and N:P molar ratios (log-transformed to ensure robust and reproducible results [65]), between birds and other vertebrate groups. We used Brown–Forsythe tests to assess equality of variances and Wilcoxon rank-sum tests to compare medians (see electronic supplementary material, table S45 for sample sizes). These non-parametric approaches were selected because several variables exhibited heteroscedasticity and non-normal distributions. To account for the increased risk of Type I errors due to multiple pairwise comparisons, p-values were adjusted using the Benjamini–Hochberg false discovery rate procedure. We compared bird stoichiometry data to other studied vertebrates using data from Andrieux et al. [8]. We also conducted the same comparisons using vertebrate data from González et al. [19]—most of which overlaps with Andrieux et al. [8]—producing nearly identical results (electronic supplementary material, figure S9). However, data comparisons with González et al. [19] are slightly more limited (e.g. only one mammal species) due to differences in data filtering. We did not combine our data with the limited existing data on the elemental composition of birds from Andrieux et al. [8] (which were grouped with reptiles in previous analyses) due to important inconsistencies in methods (e.g. feathers not included, bodies incinerated before digestion). We also excluded amphibians from our comparisons to birds due to their significant ontogenetic variability; tadpoles do not develop ossified skeletons until later stages of metamorphosis, and this differentiation is not accounted for in the existing data.
(3) *To what extent do birds vary taxonomically or phylogenetically in elemental composition?* We evaluated taxonomic differences in %C, %N and %P, as well as C:N, C:P and N:P molar ratios (log-transformed to ensure robust and reproducible results [65]), using both multivariate and univariate approaches. Specifically, we applied MANOVA to test for differences in combined elemental content and stoichiometric ratios across taxonomic groups at the species, family and order levels. For these tests, only groups with at least three replicates were included to ensure adequate statistical power (see electronic supplementary material, table S6 for sample sizes). When a MANOVA detected a significant multivariate effect (Pillai’s trace, *α* = 0.05), we conducted follow-up univariate ANOVAs for each response variable, applying Holm correction for multiple comparisons. For each univariate ANOVA, we also performed *post hoc* pairwise comparisons using Holm-adjusted pairwise tests via the *emmeans* package in R [66]. We also tested for phylogenetic signal in body elemental composition and stoichiometry using the BirdTree consensus phylogenetic tree [67] and the *phylosignal* package in R [68]. Specifically, we performed univariate analyses for each elemental variable and ratio independently to assess the degree to which trait variation correlated with phylogenetic relatedness among species. The strength of phylogenetic signal was quantified using Pagel’s λ, which ranges from 0 (no phylogenetic signal) to 1 (trait variation fully explained by phylogeny under a Brownian motion model). All elemental ratios were log-transformed before analyses to ensure robust and reproducible results [65].
(4) *What ecological and physiological factors drive variation among birds in elemental composition?* We examined the influence of body mass, diet, age, sex, fat content and feather content on the %C, %N and %P, as well as C:N, C:P and N:P molar ratios of birds (*n* = 56). Each variable was tested individually and in combination using multiple regressions with only fixed effects. We compared all models using the Akaike information criterion [69]. To meet model assumptions and capture potential allometric relationships, all elemental and stoichiometric variables were log-transformed, which improved normality and stabilized variance. We used the proportion of a species’s diet that was composed of plant material (e.g. frugivorous, granivorous) as a continuous measure of diet variability within species (electronic supplementary material, figure S7), since plant versus animal consumption reflects the most dramatic differences in diet stoichiometry and previous research shows influence of carnivory on invertebrate body stoichiometry [9]. We also tested the effects of vertebrate carnivory specifically but found no difference in our whole-body analyses. Species’ diet classifications were taken from the EltonTraits v1.0 database [51]. To normally distribute diet data and deal with zeros (i.e. a zero represents no carnivory for an obligatory frugivore), we added 10 and applied a log-transformation. Age classes were separated as ‘hatch year’ (HY) or ‘after hatch year’ (AHY) individuals based on the presence or absence of first-year plumage [70], and sex was determined based on plumage, brood patches or reproductive anatomy.

## Data Availability

All raw data are provided in the electronic supplementary material [71].
